# Human meningitis due to *Streptococcus suis* in Lomé, Togo: a case report

**DOI:** 10.1186/s12879-016-2006-0

**Published:** 2016-11-08

**Authors:** Mireille Prince-David, Mounerou Salou, Corinne Marois-Créhan, Komi Assogba, Céline Plainvert, Koffi A. Balogou, Claire Poyart, Asmaa Tazi

**Affiliations:** 1 Centre de Biologie Moléculaire et d’Immunologie, Université de Lomé, BP 101, Lomé, Togo; 2 Service de Microbiologie, Centre Hospitalier Universitaire Sylvanus Olympio, Tokoin Hôpital, BP 57, Lomé, Togo; 3 Agence nationale de sécurité sanitaire de l’alimentation, de l’environnement et du travail, 22440 Ploufragan, France; 4 Université Bretagne Loire, 22440 Ploufragan, France; 5 Service de Neurologie, Centre Hospitalier Universitaire Campus, BP 30284, Lomé, Togo; 6 Service de Bactériologie, Hôpitaux Universitaires Paris Centre, site Cochin, 75679 Paris cedex 14, France; 7 Centre National de Référence des Streptocoques, Hôpital Cochin, 75679 Paris cedex 14, France; 8 Université Paris Descartes, 75006 Paris, France

**Keywords:** Case report, *Streptococcus suis*, Meningitis, Zoonosis, Neurologic sequelae, Capsular serotype

## Abstract

**Background:**

*Streptococcus suis* is a zoonotic pathogen which represents the leading cause of meningitis in Southeast Asia and an emerging pathogen in the Western world, the main risk factor for infection being contact with pigs. In Africa, the prevalence of *S. suis* infections in swine and humans is largely unrecognized, with only one recent report of a limited case series.

**Case presentation:**

We describe a human case of meningitis due to *S. suis* in a 32-year-old man living in Togo. The patient had no particular medical history and no risk factors for immunodeficiency but reported regular contact with pork products. Using specific immunological and molecular methods, we characterized the isolate as *S. suis* serotype 2, ST1, one the most prevalent and virulent clone worldwide. The outcome was favorable after one week of adapted antibiotic therapy but the patient was left with severe hearing disorders.

**Conclusions:**

This work highlights the emergence of this pathogen in Africa and reinforces the need for accurate epidemiological and surveillance studies of *S. suis* infections and for educating clinicians and exposed groups in non-endemic countries.

## Background

*Streptococcus suis* is an emerging zoonotic pathogen in Europe, North-America and South-America [1, 2]. This encapsulated gram-positive coccus is responsible for meningitis, bacteremia, and pneumonia in swine and is mainly associated with meningitis and endocarditis in humans. Incidence is highly correlated to contact with pigs and eating of undercooked pork products, habits which are common in Southeast Asia where *S. suis* is endemic and represents the leading cause of meningitis in adults [1, 2]. In the Western world, rates of *S. suis* infection are low and the vast majority of the cases involves highly-exposed groups such as abattoir workers and pig breeders [1]. Mortality rate reaches approx. 3 % and neurologic sequelae in case of meningitis are frequent (60 %), making the global burden of *S. suis* infections in endemic countries significant [2]. We report, according to the CARE guidelines, a human case of meningitis due to *S. suis* in Africa where the prevalence of this bacterium in swine and humans is largely unrecognized.

## Case presentation

A 32-year-old man working as a carpenter in Lomé, Togo, was admitted in August 2015 to the University Hospital Campus of Lomé for a 3 day history of severe headaches, vomiting, fever, and intense agitation (Table 1). He had no particular medical history and no risk factors for immunodeficiency. At admission, the body temperature was of 36.4 °C, and the general state was acceptable, with a correct conservation of consciousness. The neurological examination did not show any sign of motor or sensitive defect, of cranial nerve dysfunction or cerebellar symptoms. There was no hearing or skin abnormalities, no obvious neck stiffness, and the rest of the clinical examination was normal. Acute meningeal hemorrhage was evocated as the initial diagnosis. Cranial computed tomography could not be performed and a symptomatic treatment associating antalgic, antipyretic, and anticonvulsant medication was initiated. At the second day of admission, the patient was febrile, agitated, and still suffered from severe headaches and vomiting. The neck had become stiff, and Kernig and Brudzinski signs were positive. The meningeal signs led to the realization of a lumbar puncture, which yielded a turbid cerebrospinal fluid (CSF) concluding to the diagnosis of bacterial meningitis. An empiric intravenous antibiotic treatment associating ceftriaxone (2 g, 2×/d for 24 h then 1 g, 2×/d), ofloxacin (200 mg, 3×/d), and metronidazole (500 mg, 2×/d) was started.

**Table 1:** Case-patient timeline

	Presenting concerns	Physical examination	Evaluations	Diagnosis	Interventions-Treatments
Day of admission	32-year-old man, carpenter in Lomé No particular medical history 3-day history of headaches, agitation, vomiting, fever	Body temperature: 36.4 °C Neurological examination: normal Clinical examination: normal	Cranial computed tomography: not performed	Acute meningeal hemorrhage	Symptomatic treatment
Day 2	Persisting symptoms Fever	Neck stiffness Kernig and Brudzinski signs positive	Lumbar puncture: turbid CSF^a^ CSF analysis: -WBC^a^: 2,800/mm3 (65 % lymphocytes,35 % neutrophils) - Protein: 1.8 g/L - Glucose: 0.47 g/L (plasma 1.41 g/L; ratio = 0.33) - Gram stain: negative	Bacterial meningitis	Wide-spectrum antibiotic therapy: ceftriaxone, ofloxacin, metronidazole
Day 3	Vertigo Tinnitus		CSF culture positive: catalase negative, gram-positive cocci in chains		Adapted antibiotic therapy: ampicillin, gentamicin Corticoid therapy
Day 4	Patient reported working as a pork butcher every weekend		Bacterial identification: *Streptococcus suis* Audiogram: severe hearing loss in left and right ears (80 dB)	Bacterial meningitis due to *S. suis* with neurological complications	
Month 9	Tinnitus		Audiogram: severe hearing loss in left and right ears (80 dB)	Neurological sequelae of the meningitis	

^a^ *CSF* cerebrospinal fluid, *WBC* white blood cells

CSF analysis showed 2,800 leukocytes/mm^3^ (65 % lymphocytes and 35 % neutrophils), a protein concentration of 1.8 g/L, and a glucose concentration of 0.47 g/L (2.6 mmol/L) with a plasma glucose concentration of 1.41 g/L (7.8 mmol/L; ratio = 0.33). Gram staining did not reveal any microorganism. Blood and CSF cultures, after 48 h and 24 h of incubation, respectively, grew small beta-hemolytic colonies on horse blood agar plates. The bacteria were catalase negative, gram-positive cocci, in chains or in pairs. They were identified as *S. suis* (score 2.56) by MALDI-TOF (Matrix Assisted Laser Desorption Ionisation Time Of Flight) spectrometry (BrukerTM) and as *S. suis* serotype 1 (97 % probability) by VITEK 2 Gram-positive card system (bioMérieux, Marcy l’Etoile, France). Slide agglutination with type-specific hyperimmune serum and specific multiplex PCR identified the isolate as *S. suis* serotype 2, indicating a serotype misidentification by the automated card system, and concluding, together with Multi-Locus Sequence Typing [3–5], to an infection due to *S. suis* serotype 2, Sequence-Type (ST) 1, one of the most virulent and frequently isolated clone all over the world [1, 6]. Antimicrobial drug-susceptibility testing performed according to the European Committee on Antimicrobial Susceptibility Testing (EUCAST) recommendations characterized the strain as susceptible to penicillin (Minimum inhibitory concentration (MIC) < =0.25 mg/L), erythromycin, clindamycin, levofloxacin, and linezolid and resistant to tetracycline.

After *S. suis* was identified, the patient reported working as a pork butcher every week-end. The patient did not have any sign or symptoms of endocarditis, and the antibiotic treatment was replaced by ampicillin (2 g, 3×/d) and gentamicin (80 mg, 2×/d) for 7 days. While the patient was receiving treatment for 1 day, vertigo and tinnitus developed, for which he received adjunctive corticoid therapy and the treatment was completed with no particular adverse events. Hearing disorders, especially hearing loss, are the most common sequelae of *S. suis* infections and affect more than 50 % of patients who survive meningitis [2]. In that case, hearing disorders evolved into severe hearing loss in the left and right ears (80 dB), and the patient was still suffering from tinnitus 9 months after the diagnosis. One year later, tinnitus had disappeared and the patient’s auditory function had improved from severe to moderate hearing loss.

## Conclusions

Data for circulation and epidemiology of *S. suis* in Africa remain extremely scarce. This is the second report of a human *S. suis* meningitis in Togo, which, together with a recently published limited case-series [7], highlights the emergence of this zoonotic pathogen in Africa. Nevertheless, swine industry and pork products consumption, although extremely low compared to other continents, are steadily increasing [8], which might lead to a significant rise of *S. suis* infections in the future. Besides, *S. suis* infections are not limited to adult cases but also affect children [7], and specific attention should be paid to this other potential high-risk group. This case-report, for which clinical data, microbiological findings, and outcome were similar to those reported elsewhere [1, 2], emphasizes the need for educating both clinicians and exposed groups about *S. suis* infections and their severity in non-endemic countries. Particularly, accurate epidemiological and surveillance studies appear essential to assess the global burden of *S. suis* infections in swine and humans in Africa.

## Abbreviation

CSF: Cerebrospinal fluid
